# Short 2-[^18^F]Fluoro-2-Deoxy-D-Glucose PET Dynamic Acquisition Protocol to Evaluate the Influx Rate Constant by Regional Patlak Graphical Analysis in Patients With Non-Small-Cell Lung Cancer

**DOI:** 10.3389/fmed.2021.725387

**Published:** 2021-11-22

**Authors:** Luca Indovina, Valentina Scolozzi, Amedeo Capotosti, Stelvio Sestini, Silvia Taralli, Davide Cusumano, Romina Grazia Giancipoli, Gabriele Ciasca, Giuseppe Cardillo, Maria Lucia Calcagni

**Affiliations:** ^1^Fondazione Policlinico Universitario A. Gemelli IRCCS, Rome, Italy; ^2^Unità Operativa Complessa (UOC) di Medicina Nucleare, Dipartimento di Diagnostica per Immagini, Radioterapia Oncologica ed Ematologia, Fondazione Policlinico Universitario A. Gemelli IRCCS, Rome, Italy; ^3^Nuclear Medicine Unit, NOP S. Stefano, Prato, Italy; ^4^Università Cattolica del Sacro Cuore, Rome, Italy; ^5^Unit of Thoracic Surgery, San Camillo Forlanini Hospital, Rome, Italy; ^6^Dipartimento Universitario di Scienze Radiologiche ed Ematologiche, Università Cattolica del Sacro Cuore, Rome, Italy

**Keywords:** PET dynamic acquisition, Patlak graphical analysis, non-small-cell lung cancer, influx rate constant, 2-[^18^F]Fluoro-2-deoxy-D-glucose

## Abstract

**Purpose:** To test a short 2-[^18^F]Fluoro-2-deoxy-D-glucose (2-[^18^F]FDG) PET dynamic acquisition protocol to calculate K_i_ using regional Patlak graphical analysis in patients with non-small-cell lung cancer (NSCLC).

**Methods:** 24 patients with NSCLC who underwent standard dynamic 2-[^18^F]FDG acquisitions (60 min) were randomly divided into two groups. In group 1 (*n* = 10), a population-based image-derived input function (pIDIF) was built using a monoexponential trend (10–60 min), and a leave-one-out cross-validation (LOOCV) method was performed to validate the pIDIF model. In group 2 (*n* = 14), K_i_ was obtained by standard regional Patlak plot analysis using IDIF (0–60 min) and tissue response (10–60 min) curves from the volume of interests (VOIs) placed on descending thoracic aorta and tumor tissue, respectively. Moreover, with our method, the Patlak analysis was performed to obtain K_i,s_ using IDIF_Fitted_ curve obtained from PET counts (0–10 min) followed by monoexponential coefficients of pIDIF (10–60 min) and tissue response curve obtained from PET counts at 10 min and between 40 and 60 min, simulating two short dynamic acquisitions. Both IDIF and IDIF_Fitted_ curves were modeled to assume the value of 2-[^18^F]FDG plasma activity measured in the venous blood sampling performed at 45 min in each patient. Spearman's rank correlation, coefficient of determination, and Passing–Bablok regression were used for the comparison between K_i_ and K_i,s_. Finally, K_i,s_ was obtained with our method in a separate group of patients (group 3, *n* = 8) that perform two short dynamic acquisitions.

**Results:** Population-based image-derived input function (10–60 min) was modeled with a monoexponential curve with the following fitted parameters obtained in group 1: *a* = 9.684, *b* = 16.410, and *c* = 0.068 min^−1^. The LOOCV error was 0.4%. In patients of group 2, the mean values of K_i_ and K_i,s_ were 0.0442 ± 0.0302 and 0.33 ± 0.0298, respectively (*R*^2^ = 0.9970). The Passing–Bablok regression for comparison between K_i_ and K_i,s_ showed a slope of 0.992 (95% CI: 0.94–1.06) and intercept value of −0.0003 (95% CI: −0.0033–0.0011).

**Conclusions:** Despite several practical limitations, like the need to position the patient twice and to perform two CT scans, our method contemplates two short 2-[^18^F]FDG dynamic acquisitions, a population-based input function model, and a late venous blood sample to obtain robust and personalized input function and tissue response curves and to provide reliable regional K_i_ estimation.

## Introduction

The 2-[^18^F]Fluoro-2-deoxy-D-glucose (2-[^18^F]FDG) Positron Emission Tomography/Computed Tomography (PET/CT) is a well-established imaging modality for staging, restaging, and monitoring treatment response in patients with malignancy (1–3). Absolute quantification of 2-[^18^F]FDG concentration, related to the local metabolic rate of glucose consumption measured with full kinetic analysis of time-activity curves, has proven to better characterize the tumor cell behavior and to correlate with histopathological data and prognosis (4–6). Nevertheless, some key points of full kinetic analysis, mainly the long dynamic acquisition and arterial blood sampling, limit the use of such an approach in a clinical setting. For these reasons, less-invasive approaches, including measurements of semiquantitative parameters such as the most common standard uptake value (SUV), are extensively used routinely (7–9). However, it has shown that the accuracy of SUV depends on several factors, including the standardization of technical parameters (e.g., acquisition protocol, glucose blood level, scan time window, recovery coefficient, partial volume effect, region-of-interest definition, and different PET scanners), which can affect the reliability of uptake values (10–13).

The Patlak graphical analysis is a valid alternative to full kinetic analysis for radioligands with irreversible kinetics as 2-[^18^F] FDG since the activity of the phosphatases is considered negligible (14). The Patlak analysis provides the value of the influx rate constant K_i_ [min^−1^] related to the metabolic rate of glucose in tissue. The K_i_ parameter is calculated from the slope of a straight line that correlates the integral radioligand activity in the blood pool with radioligand activity in the tissue (15). For this purpose, a long PET dynamic acquisition lasting at least 60 min is considered mandatory to calculate the time-activity curves of 2-[^18^F]FDG in the blood (input function) and tissue (tissue response). Several efforts have been made to simplify the input function estimation with less- or non-invasive methods, such as the arterialized venous blood sampling (16), the image-derived input function (IDIF) estimation (17–20), the population input function modeling (21–24), the image segmentation methods (25, 26), and to overcome difficulties related to the long-lasting dynamic acquisition (27–30).

To reduce the dynamic 2-[^18^F]FDG PET acquisition time (28–30), the aim of this study was to test a short dynamic protocol to obtain the input function and the tissue response curves to calculate K_i_ using the Patlak analysis and to compare it with K_i_ obtained using the standard long dynamic acquisition protocol in patients with non-small cell lung cancer (NSCLC).

## Materials and Methods

### Patients

24 patients (15 male patients, mean age 69 ± 11 years) with histologically proven NSCLC, referred for staging to PET/CT center of Fondazione Policlinico Universitario A. Gemelli IRCCS in Rome by the local Thoracic Surgery Unit of San Camillo Forlanini Hospital, were enrolled. All patients (*n* = 24) underwent standard long (0–60 min) dynamic PET acquisition over the thorax followed by a total body scan. Patients were randomly divided into two groups: group 1 (*n* = 10; six male patients, mean age 70 ± 12 years) was used to extrapolate and validate the population-based image-derived input function (pIDIF); group 2 (*n* = 14; nine male patients, mean age 69 ± 10 years) was used to compare K_i_ obtained with our method (simulating two short dynamic acquisition and using the pIDIF validated in group 1) with K_i_ obtained using the standard long dynamic acquisition. Finally, a separate third group of patients (*n* = 8; seven male patients, mean age 71 ± 11 years) with histologically proven NSCLC were enrolled to test the feasibility of our method in clinical practice. All patients (*n* = 8) underwent two separate short dynamic PET acquisitions over the thorax followed by a total body scan. The local institution's ethics committee (Comitato Etico Lazio 1) approved this retrospective study, waiving written informed consent for participation.

### 2-[^18^F]FDG PET/CT: Acquisition and Reconstruction Parameters

All patients were fasted for at least 6 h and in normoglycemic (glucose level <150 mg/dl) conditions before PET acquisition. PET/CT studies were performed using a full-ring CT and PET-integrated tomograph (3D Biograph mCT, Siemens Healthineers, Chicago, Illinois). Patients were placed in a supine position with the thorax in the field of view and the arms placed over the head. The acquisition protocol started with a CT scout including the thoracic aorta and lungs. A low-dose CT was performed (90 mA, 120 kV) for the attenuation correction of emission data and morphological information with a field of view of 21 cm. The transaxial CT matrix size was 512 × 512 (1 × 1 × 3 mm).

#### Standard Long Dynamic Acquisition

Patients of groups 1 and 2 were intravenously injected with 134–507 MBq of 2-[^18^F]FDG, using an infusion pump (model RADInject; Tema Sinergie, Faenza, RA, Italy); 10 ml of 2-[^18^F]FDG was administered at a rate of 4.32 ml/s followed by a 10-ml saline flush. After 2-[^18^F]FDG injection, a thorax dynamic list-mode acquisition lasting 60 min was started with the following framing: 24 frames of 5 s each, 12 frames of 15 s each, and 11 frames of 5 min each. A venous blood sampling was performed at 45 min post-injection. Dynamic PET data were corrected for random events, dead time, and attenuation. PET data were reconstructed with the ordered subset expectation maximization (OSEM) algorithm, including time-of-flight and UltraHD recovery with 21 subsets and two iterations. The transaxial PET matrix size was 256 × 256 (3.18m × 3.18 × 3 mm).

#### Two Short Dynamic Acquisitions

Patients of group 3 were intravenously injected with 205–320 MBq of 2-[^18^F]FDG using the same infusion pump and protocol of groups 1 and 2. After 2-[^18^F]FDG injection, early thorax dynamic list-mode acquisition lasting 10 min was started with the following framing: 24 frames of 5 s each, 12 frames of 15 s each, and one frame of 5 min each; late thorax dynamic list-mode acquisition lasting 20 min was started at 40 min post-injection with the following framing: four frames of 5 min each. Two low-dose CTs were performed for each acquisition to assess an accurate attenuation correction of the two PET images. Patients left the examination PET/CT room in the period between early and late PET/CT examinations. A venous blood sampling was performed at 45-min post-injection. The same algorithm and reconstruction parameters of the standard long dynamic acquisition were used.

### Input Function

In each patient of group 1 (*n* = 10), the standard input function was obtained drawing a volume of interest (VOI) on the descending thoracic aorta during the summed first nine frames of dynamic acquisition (45 s) and superimposing it on all subsequent frames of dynamic acquisition (0–60 min) (31). For each patient, the venous blood sampling performed at 45 min after injection was centrifuged for 5 min (Rotofix 32A; Hettich Italia S.r.l., Milano, Italy) to separate the plasma from the cellular components. The 2-[^18^F]FDG activity in 1 ml of plasma was measured in a gamma counter (Wallac Wizard 1480−3″; PerkinElmer, Waltham, Massachusetts) cross-calibrated with the tomograph. The IDIF curve was modeled to assume the value of 2-[^18^F]FDG activity measured in the plasma (32); in particular, a scale factor equal to the ratio between the plasma activity measured in 1 ml of plasma, and the activity measured in the IDIF at 45 min was used to impose the IDIF activity value at 45 min equal to the plasma activity measured in the blood sample, considering the plasma activity as the gold standard value and avoiding any problem regarding spillover effect or partial volume effect in the VOI signal.

To extrapolate and validate the input function used in our method, it was reconstructed taking into account only data from 0 to 10 min of the standard long dynamic acquisition, simulating an early short dynamic acquisition (pIDIF). In particular, for each patient of group 1 (*n* = 10), the early phase of the input function was built taking into account the first 10 min of the patient's input function curve of the standard long dynamic acquisition. The remaining part was reconstructed with a monoexponential function (23, 33) since all input function curves obtained from standard dynamic protocol showed a trend that can be well represented by a monoexponential function (1):


(1) $$\begin{array}{r} C_{p}\left(t\right)=a+b\cdot e^{\left(-c\cdot t\right)} \end{array}$$

where *a, b*, and *c* coefficient values were obtained as mean values of monoexponential fit of each 10 IDIF, measured from 10 to 60 min in patients of group 1.

Then, a specific patient input function was built imposing the pIDIF 2-[^18^F]FDG activity value at 45 min equal to the plasma activity in each patient measured at 45 min from the venous sample (IDIF_Fitted_).

Lastly, the LOOCV method was performed to validate the method of pIDIF reconstruction.

### K_i_ Estimation Using the Standard Dynamic Protocol

The K_i_ parameter was estimated using the following formula (2):


(2) $$K_{i}=\frac{C_{T}(T)}{\int_{0}^{T}C_{p}(t)\cdot dt}-V_{D}\cdot \frac{C_{p}(T)}{\int_{0}^{T}C_{p}(t)\cdot dt}$$

where K_i_, is the influx rate constant, *C*_T_(*t*) is the mean value for the radioligand concentration in tissue during the time (tissue response), *T* is the time of dynamic acquisition, *V*_D_ is the distribution volume, *C*_p_(*t*) is the radioligand concentration in plasma during the time (i.e., the IDIF), and the integral symbol represents the area under the curve of the IDIF. Since both C_T_ and C_p_ are obtained from the VOI in tumor tissue and in descending thoracic aorta, respectively, the proposed analysis is based on regional and not voxel-based Patlak parametric imaging method.

For each patient of group 2 (*n* = 14), K_i_ was estimated with the Patlak analysis using the IDIF (0–60 min) and the tissue response (10–60 min) curves. The IDIF was obtained as described in the previous paragraph. The tissue response curve was obtained by drawing VOI on the tumor in the last frame of the dynamic acquisition and superimposing it on all previous frames (10–60 min). VOIs for the input function and tissue response were automatically placed over three consecutive slices to include the five hottest pixels within the VOI (34, 35).

### K_i_ Estimation Simulating the Short Dynamic Protocol (K_i,s_)

For each patient of group 2 (*n* = 14), K_i_ was estimated using data from 0 to10 min and from 40 to 60 min of the long standard acquisition to obtain the IDIF_Fitted_ and the tissue response curves simulating two short dynamic acquisitions.

In particular, for each patient, the early phase of the input function was built taking into account the first 10 min of the patient's input function curve of the standard dynamic protocol. The remaining part was reconstructed with the previous reported monoexponential function (1) using the *a, b*, and *c* coefficients of pIDIF obtained and validated in patients of group 1, then modeled to assume at 45 min the value of 2-[^18^F]FDG activity in the plasma measured at 45 min from the venous sample (IDIF_Fitted_), as previously described.

For each patient, the tissue response curve was reconstructed taking into account data measured at 10 min and those measured between 40 and 60 min of the patient's tissue response curve of the standard protocol.

Finally, the influx rate constant (K_i,s_) was estimated with the Patlak analysis according to the well-known formula (2) using the IDIF_Fitted_ and the tissue response curves as described.

### K_i_ Estimation Using the Short Dynamic Protocol (K_i,s_)

For each patient of group 3 (*n* = 8), *K*_i_ was estimated using two short dynamic acquisitions performed to obtain the IDIF_Fitted_ and the tissue response curves.

In particular, the IDIF_Fitted_ was built drawing a VOI on the descending thoracic aorta during the summed first nine frames of early dynamic acquisition (45 s) and superimposing it on all subsequent frames of the early dynamic acquisition (0–10 min); the remaining part (10–60 min) was reconstructed using the *a, b*, and *c* coefficients values of pIDIF obtained and validated in patients of group 1, then modeled to assume at 45 min the value of 2-[^18^F]FDG activity in the plasma measured at 45 min from the venous sample (IDIF_Fitted_), as previously described.

The tissue response curve was obtained drawing a VOI on the tumor in the last frame of the late dynamic acquisition and superimposing it on all previous frames (40–60 min); another VOI was drawn on the tumor in the last frame of the early dynamic examinations (10 min). VOIs for input function and tissue response were automatically placed over three consecutive slices to include the five hottest pixels within the VOI (34, 35). Being a VOI-based analysis method and therefore limited to specific regions (not voxel-wise), the co-registration between early and late PET images was not necessary.

Finally, the influx rate constant (K_i,s_) was estimated using the Patlak analysis according to the well-known formula (2) using the IDIF_Fitted_ and the tissue response curves as described.

### Standardized Uptake Value

Standard uptake value was calculated as following formula (3):


(3) $$\begin{array}{r} SUV=\frac{{\text{C}}_{\text{T}}\left(T\right)}{A_{0}/bw} \end{array}$$

where *C*_T_(T) is the value of radioligand concentration at T equal to 55-min post-injection measured using a VOI drawn on tumor tissue (tissue response) in the last frame, *A*_0_ is the injected activity, and bw is the bodyweight of the patient (36).

### Statistical Analysis

R (analytical software) was used for calculations. The mean square percentage error (MSEP) between standard IDIF (0–60 min) and IDIF_fitted_ was calculated, and LOOCV_error_ was computed to perform the leave-one-out cross-validation. The concordance between K_i_ and K_i,s_ was evaluated using the Passing–Bablok regression. The Spearman's rank correlation coefficient (ρ) and coefficient of determination (*R*^2^) were used to assess the correlation between K_i_ estimated using standard dynamic protocol and K_i,s_ estimated with short dynamic protocol, and between K_i,s_ and SUV.

## Results

**Figure 1:**
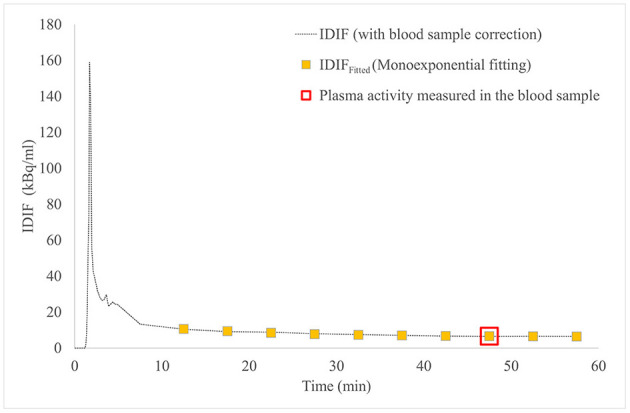
IDIF curve (dashed line) and IDIF_Fitted_ curve (yellow square boxes), both modeled to pass through the 2-[^18^F]FDG activity in plasma measured in the venous blood sample at 45 min post-injection (red square box), in patient #4. 2-[^18^F]FDG, 2-[^18^F]Fluoro-2-deoxy-D-glucose; IDIF, image-derived input function.

In 24 patients with NSCLC who performed the standard long dynamic protocol (groups 1 and 2), the input function curves showed a monoexponential trend from 10 min after 2-[^18^F]FDG injection up to the end of acquisition (60 min), as reported in a representative patient (Figure 1).

The mean values of *a, b*, and *c* coefficients obtained from monoexponential fittings of 10 input function curves (group 1) were 9.684, 16.410, and 0.068 min^−1^, respectively.

Figure 1 shows IDIF obtained with standard long dynamic protocol and IDIF_Fitted_ obtained with short dynamic protocol and reconstructed with monoexponential fit in a representative patient (#4): both curves were imposed to pass through the measured 2-[^18^F]FDG activity in plasma at 45 min.

The LOOCV_error_ between standard IDIF (0–60 min) and IDIF_fitted_ was 0.4%.

Table 1 reports demographic data and PET parameters values (K_i_, K_i,s_, SUV) for all patients of group 2 (*n* = 14).

**Table 1:** Demographic data and PET parameters values in patients of group 2 (*n* = 14).

**Patients**	**Age**	**Sex**	**K_i_**	***R*** **^2^ (K_i_)**	**K_i,s_**	***R*** **^2^ (K_i,s_)**	**SUV**
2	75	M	0.0314	0.9838	0.0291	0.9748	7.69
4	74	M	0.0381	0.9900	0.0377	0.9873	11.28
6	73	M	0.0385	0.9475	0.0361	0.9808	7.86
9	77	M	0.0468	0.9820	0.0485	0.9833	14.32
11	73	F	0.0403	0.9983	0.0418	0.9965	10.29
13	70	M	0.0383	0.9572	0.0349	0.9545	8.75
15	74	F	0.0222	0.9342	0.0222	0.9364	9.10
18	72	F	0.0165	0.9812	0.0166	0.9839	5.88
21	81	M	0.0310	0.9640	0.0290	0.9593	5.97
24	40	F	0.0303	0.9952	0.0313	0.9953	5.83
25	74	M	0.0764	0.9813	0.0733	0.9859	15.20
26	61	M	0.0656	0.9906	0.0654	0.9951	11.60
27	64	M	0.1306	0.9941	0.1287	0.9936	25.92
31	58	F	0.0123	0.9782	0.0117	0.9877	4.46

*K_i_, influx rate constant obtained with the Patlak graphical analysis using the standard long dynamic protocol; R^2^ (K_i_), Patlak plot linear regression coefficient for K_i_; K_i,s_, influx rate constant obtained with the Patlak graphical analysis using the short dynamic protocol; R^2^ (K_i,s_), Patlak plot linear regression coefficient for K_i,s_; SUV, standardized uptake value*.

The mean values (±SD) of K_i_ obtained with standard long dynamic protocol and K_i,s_ obtained simulating the short dynamic protocol for patients of group 2 (*n* = 14) were 0.0442 min^−1^ (±0.0302) and 0.0433 min^−1^ (±0.0298), respectively. The correlation coefficient and coefficient of determination between the two parameters were ρ = 0.974 and *R*^2^ = 0.9970, respectively.

**Figure 2:**
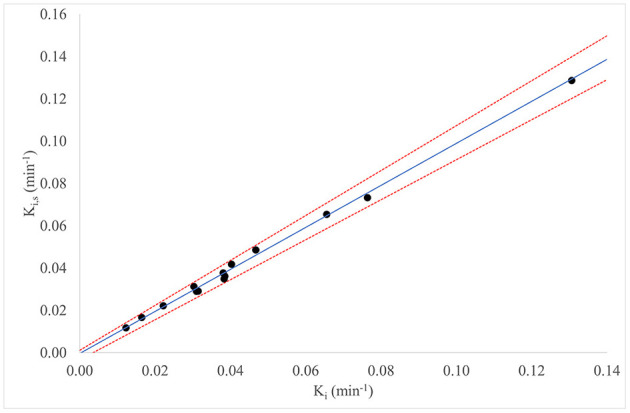
Passing–Bablok regression for comparison of K_i_ and K_i,s_, for all patients in group 2 (*n* = 14).

Figure 2 reports for all patients of group 2 (*n* = 14) the Passing–Bablok regression for the comparison between K_i_ and K_i,s_: the comparison between the two methods showed a slope value of 0.992 (95% CI: 0.94–1.06) and intercept value of −0.0003 (95% CI: −0.0033 to 0.0011).

Table 2 reports demographic data and PET parameters values (K_i,s_, SUV) for all patients in group 3 (*n* = 8).

**Table 2:** Demographic data and PET parameters values in patients of group 3 (*n* = 8).

**Patients**	**Age**	**Sex**	**K_i,s_**	**R^2^ (K_i,s_)**	**SUV**
1	64	M	0.0646	0.9985	11.56
2	80	M	0.1018	0.9915	19.64
3	76	M	0.0374	0.9920	9.26
4	73	M	0.1188	0.9954	19.89
5	84	M	0.0614	0.9939	12.61
6	71	F	0.0697	0.9902	15.35
7	72	M	0.0485	0.9446	7.15
8	47	M	0.0971	0.9183	14.96

*K_i,s_, influx rate constant obtained with the Patlak graphical analysis using the short dynamic protocol; R^2^(K_i,s_), Patlak plot linear regression coefficient for K_i,s_; SUV, standardized uptake value*.

**Figure 3:**
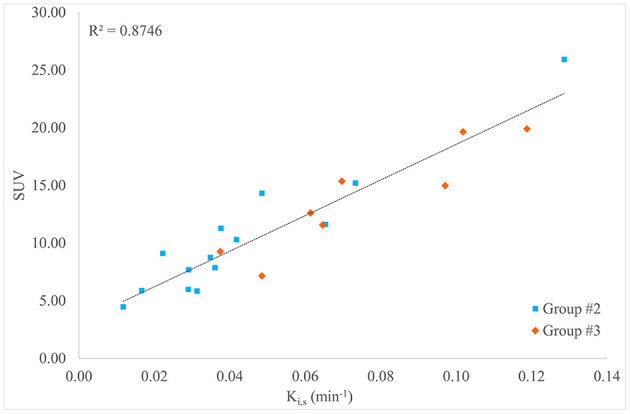
Scatter plot, linear regression, and *R*^2^ value for the comparison between K_i,s_ and standard uptake value (SUV) for all patients in groups 2 and 3 (*n* = 22).

In patients of group 2 (*n* = 14, standard long dynamic acquisition) and those of group 3 (*n* = 8, two short dynamic acquisitions), the overall mean value (±SD) of SUV of the primary tumor was 11.57 (±5.37). The correlation coefficient and coefficient of determination between SUV and K_i,s_ values were ρ = 0.923; *R*^2^ = 0.8746 (*n* = 22), respectively, as reported in Figure 3.

**Figure 4:**
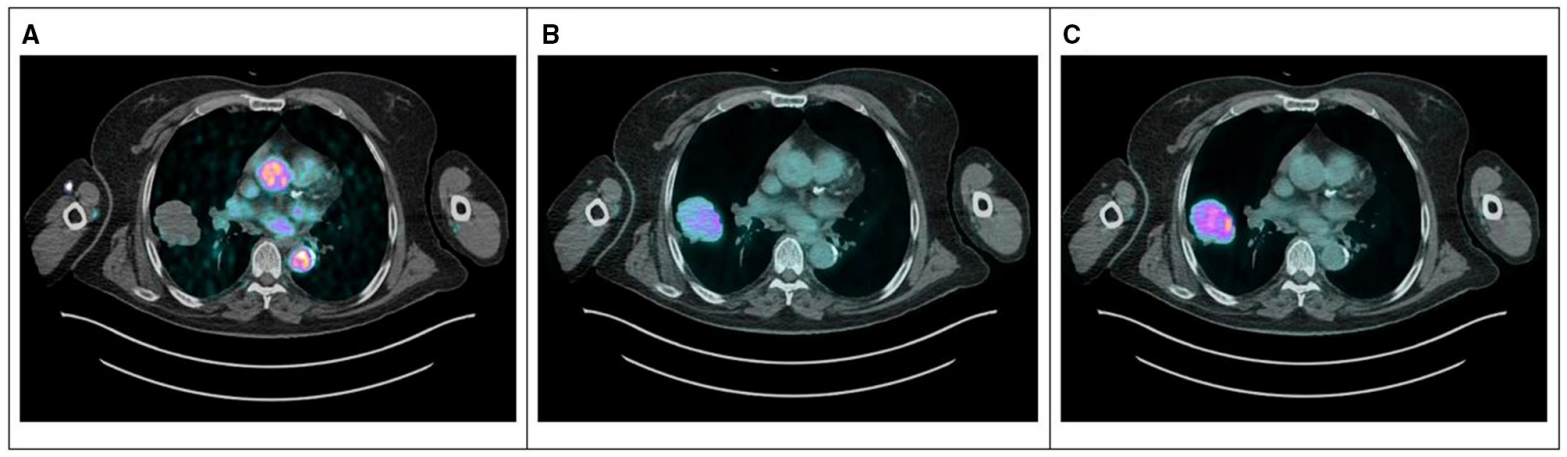
PET/CT images at 2 min **(A)**, 30 min **(B)**, and 60 min **(C)** after 2-[^18^F]FDG injection for a representative patient (#4) showing the blood pool in the early phase and the increasing uptake in the lesion during the time. 2-[^18^F]FDG, 2-[^18^F]Fluoro-2-deoxy-D-glucose.

Figure 4 shows 2-[^18^F]FDG uptake at 2 min (Figure 4A), 30 min (Figure 4B), and 60 min (Figure 4C) in the thoracic aorta and primary tumor after tracer injection in a representative patient (#4).

## Discussion

The Patlak graphical analysis has been used in dynamic PET to estimate the influx rate constant (K_i_) of tracers with irreversible uptake including 2-[^18^F]FDG (14, 15, 37). Among several quantification methods, the Patlak analysis is a reliable and robust approach providing an accurate measure of K_i_ that is less affected by technical parameters and by tissue heterogeneity (38, 39).

Nevertheless, the kinetics of 2-[^18^F]FDG requires a long dynamic PET acquisition lasting at least 60 min with consequent discomfort for the patient, limiting its use in clinical routine (40). To overcome such limitations, in this study a shorter dynamic acquisition protocol has been proposed to estimate K_i_ by Patlak analysis in patients with NSCLC. The input function and the tissue response curves were reconstructed taking into account data (0–10 min and 40–60 min) from standard long dynamic acquisition, simulating two short dynamic PET acquisitions.

Regarding input function, it is well known that its accurate determination is a key point for kinetic modeling. The Patlak analysis requires the knowledge of the full course of input function time–activity curve, from the tracer injection to the end of dynamic acquisition of 60 min (41). In our method, the first part of each input function curve was built using data obtained from injection up to 10 min of the standard long dynamic acquisition, simulating a short early dynamic acquisition. The remaining part was reconstructed with monoexponential fitting using the mean value of *a, b*, and *c* coefficients obtained from standard input function values of a separate group of patients. Indeed, we observed that all curves of the standard long dynamic acquisition showed a monoexponential trend from the 10-min post-injection to the end. Finally, each input function curve so obtained was modeled to assume the value of 2-[^18^F]FDG activity measured in the venous blood sample at 45 min post-injection. Indeed, at late times, an equilibrium between 2-[^18^F]FDG concentration in arterial and venous blood is reached (35, 42). Moreover, the radioligand activity measured in blood better represents the “real” 2-[^18^F]FDG concentration compared to that measured using only VOI. The reliability of this method is supported by the very low LOOCV_error_ value between standard IDIF and IDIF_fitted_. Therefore, a short dynamic acquisition plus a late venous blood sample can be sufficient to construct a robust input function that can be considered “personalized” since it uses data that are, largely, patients own data (counts in first 10 min of dynamic acquisition and counts in a venous blood sample) plus reconstructed data (monoexponential fitting). Therefore, we underline that the input function curve has to be as accurate as possible taking also into account the physio- and pathological characteristics of each patient representing the “true” tracer bioavailability. Regarding the tissue response, it was built using data obtained from 40 to 60 min of the standard long dynamic acquisition, simulating a late-short dynamic acquisition, plus counts of the last frame (10 min) of the simulated early-short dynamic acquisition, useful to well estimate the slope of Patlak plot.

Finally, the reliability of K_i_,_s_ values calculated using the input function and tissue response curves obtained with the short protocol is supported by the high correlation with K_i_ obtained with the standard long dynamic acquisition. This finding suggests the feasibility of the short protocol in clinical practice, requiring only two short dynamic PET acquisitions (0–10 min and 40–60 min) instead of the longer standard one (60 min), plus a late venous blood sampling. In addition, such a method that contemplates two dynamic acquisitions (one including a large vessel for input function; the other including the neoplastic lesion for tissue response) can be applied to evaluate the influx rate constant (and other quantitative parameters) of neoplastic lesion located in everybody site. Recently, Wu et al. (43) investigated the feasibility of generating K_i_ for 2-[^18^F]FDG PET from dual-time-point imaging data (5 min per scan) by using a population-based input function. Differently from our study, they did not extrapolate K_i_ from a linear regression but the angular coefficient of a straight line passing through two points. Moreover, the scaling factor for the population-based curve was determined on the summation of the input function values at the middle time points of the early and the late scans.

Regarding semiquantitative PET parameters, SUV showed a good (even if not excellent) correlation with K_i,s_ obtained both in patients in whom the two short dynamic acquisitions were simulated and in patients in whom two short dynamic acquisitions were performed. This result confirms that semiquantitative parameters, even if less accurate, well represent the glucose metabolism only if all technical and procedural aspects in patient preparation and scan acquisition are strictly respected. Indeed, especially when several PET/CT examinations are repeated over time for the evaluation of response to oncological treatment (44–46), the difficulty in strictly respecting all technical aspects can make the semiquantitative parameters unreliable. Moreover, it is important to remind that the accuracy of semiquantitative parameters may be affected by non-controlled aspects such as the oncological therapies that can modify the tracer bioavailability (especially the news antiangiogenic drugs), the cancer cells biological characteristics (tracer uptake), and other unknown biologic and patients factors (47, 48). Furthermore, we have to take into account that semiquantitative parameters, such as SUV, perform relatively poorly when the tumor-to-background ratio is low as in liver lesions, in small and less 2-[^18^F]FDG avid tumors (49) or post-treatment evaluation when the uptake in tumors may be suppressed after therapy (19). Therefore, the use of kinetic parameters seems preferable to semiquantitative ones, not only for therapy monitoring (50–52) but also for evaluating glucose metabolism of tumor regions with relatively high background activity (19). Moreover, it was recently showed that oncologic whole-body (WB) Patlak K_i_ imaging may improve lesion detectability reducing false-positive rates when complementing SUV (53). This potential improvement in specificity may support the use of kinetic parameters in other clinical settings, such as for differential diagnosis between pulmonary tumors and inflammatory lesions, and between progression and pseudoprogression during immunotherapy.

Regarding practical aspects of our method, to come in and out of the PET scanner twice to perform two separate acquisitions did not determine a discomfort for patients of group 3 who performed the short dynamic protocol. Indeed, the short lasting of PET acquisitions (compared to the long standard one) seems preferable for patients who have to maintain the correct position with the arms over the head on the scanner for a shorter time. In addition, the method does not require a rigid repositioning of the patient on the PET scanner, since a co-registration of early and late dynamic images was not needed. However, this could be a limitation if a voxel-wise Patlak analysis method would be explored; in that case, a similar approach may not be feasible due to the need for co-registration between early and late dynamic PET frames. Finally, this procedure that requires two separate short acquisitions, between which the patient leaves the scanner free for other patients PET acquisition, may result in several practical limitations, increasing the complexity in the scheduling of the daily clinical workflow, the time involvement of the staff in repositioning the patient on the scanner table, and the risk of propagating delay or cancellations of one exam on the following ones. Moreover, we have to take into account that the need for two low-dose CT scans for accurate attenuation correction of the two dynamic PET acquisitions introduces additional radiation exposure for the patient and increases the time involvement for patients and staff, reducing the time between the early and late acquisitions in which the scanner is available for another exam. However, this proposed method cannot replace the standard-of-care WB PET acquisitions, but it can allow quantifying K_i_ in few selected cases (maximum three per day for clinical or research aims), even in PET centers that do not have advanced technologies, scheduling them at the beginning of the end of the daily workflow, reducing the impact on daily clinical activities as much as possible. However, we have to take into account that there are currently commercially available products that provide fully automated WB parametric PET images. Their diffusion in the next future will allow acquiring dynamic WB PET studies with the arms in the down position, improving the comfort of patients and limiting the possibility of motion-induced artifacts in the PET images, with minimal control and time involvement requirements for the staff.

Beyond the practical aspects, the main limitations of this study are the relatively small sample size, the absence of arterial blood samples as the reference standard (being invasive and not feasible in clinical practice) to validate the input function time–activity curve, and the application of the short protocol only in patients with NSCLC. Moreover, the application of a single-scale factor to impose the IDIF activity value at 45 min equal to the plasma activity measured in the blood sample could be a potential limitation due to the different partial volume effects during time scan; in the early phase, the activity in the vessel is very high causing spill-out of activity outside the vessel wall boundaries and lead to a possible underestimation of the IDIF peak. On the contrary, in the late phase, the activity inside the vessel is expected to be very small, whereas the activity from the surrounding tissue is relatively larger, thus causing a possible overestimation of the IDIF value. In addition, the use of two short dynamic acquisitions could not be adequate to apply the generalized Patlak methods developed and employed in K_i_ quantification when considering a mild degree of reversibility of 2-[^18^F]FDG kinetics, since these methods require multiple measurements at both early and late time points (23, 54–56). Nevertheless, the robustness of the performance of our method in the K_i_ evaluation using two short acquisitions (compared to the standard one) supports its use, aware that with the simplicity of our method, a good estimate of the value of the constant influx rate was obtained. Finally, the use of 4D reconstruction algorithms (30, 57–59), recently available in some PET scanners, but not applied in our PET scans, would mitigate statistical noise levels, improving image quality and K_i_ estimation.

## Conclusion

In conclusion, our proposed method may provide a reliable quantification of regional estimates of influx rate constant for tissues not expressing 2-[^18^F]FDG uptake reversibility. Two short dynamic PET acquisitions obtained at an early and late time point post-injection plus a population-based input function model scaled according to a late venous blood sample may be enough to obtain a robust and personalized temporal integral of the input function, which is necessary to estimate the net influx rate constant by regional Patlak analysis. This short dynamic protocol of two scan sessions at 0–10 min and 40–60 min post-injection may have some potential advantages when compared with the standard dynamic WB long 60-min acquisition protocols; it can reduce the total time spent inside the scanner for each patient, but not its total exam time involvement. The scan time reduction could therefore mitigate their discomfort for some patients if exiting and entering the scanner twice per exam is not an issue; however, this reduction in acquisition time comes at the cost of additional radiation exposure for the patient with a second low-dose WB CT scan. Despite several practical limitations, such as the increase in CT radiation dose from the need for a second low-dose WB CT exam, the complexity of scheduling daily exams with interleaved sessions between different patients and the risk of propagating time delays and other problems from one exam session to the exams of other patients in the same day, the proposed dynamic PET/CT scan protocol can theoretically allow performing more dynamic PET acquisitions daily; furthermore, similarly to other WB dynamic PET scan protocols, it allows to obtain regional estimates of highly quantitative parameters in tumor regions located in distant organs scanned at different bed positions. Moreover, data analysis is not more time-consuming and does not require additional expertise compared to other dynamic WB PET/CT protocols. From the clinical point of view, the use of dynamic WB PET acquisitions assumes more significance in oncological patients in whom the quantification is more relevant than semiquantification, especially in treatment monitoring and prognostic assessment. Our proposed method, along with other recent dynamic WB PET/CT studies (28, 29, 43, 58), aims to facilitate the clinical adoption of dynamic PET and regional parametric analysis by shortening the total PET scan times often required in these protocols.

## Data Availability Statement

The raw data supporting the conclusions of this article will be made available by the authors, without undue reservation.

## Ethics Statement

The studies involving human participants were reviewed and approved by Comitato Etico Lazio 1. The ethics committee waived the requirement of written informed consent for participation.

## Author Contributions

MC and LI contributed to the conception and design of the study. VS, ST, RG, and GCa were involved in the acquisition of PET/CT and clinical data. LI and AC performed data analysis. LI, VS, AC, SS, DC, GCi, and MC were involved in data interpretation. LI, VS, AC, and SS drafted the manuscript. MC critically revised the manuscript for important intellectual content. All authors revised the final manuscript and gave their final approval for the manuscript submission.

## Conflict of Interest

The authors declare that the research was conducted in the absence of any commercial or financial relationships that could be construed as a potential conflict of interest.

## Publisher's Note

All claims expressed in this article are solely those of the authors and do not necessarily represent those of their affiliated organizations, or those of the publisher, the editors and the reviewers. Any product that may be evaluated in this article, or claim that may be made by its manufacturer, is not guaranteed or endorsed by the publisher.
